# Growth of SnO_2_ Nanoflowers on N-doped Carbon Nanofibers as Anode for Li- and Na-ion Batteries

**DOI:** 10.1007/s40820-017-0172-2

**Published:** 2017-12-08

**Authors:** Jiaojiao Liang, Chaochun Yuan, Huanhuan Li, Kai Fan, Zengxi Wei, Hanqi Sun, Jianmin Ma

**Affiliations:** 1 School of Physics and Electronics, Hunan University, Changsha, 410082 People’s Republic of China; 2 Automotive Engineering Research Institute, Jiangsu University, Zhenjiang, 212013 People’s Republic of China; 3 Key Laboratory of Advanced Energy Materials Chemistry (Ministry of Education), Nankai University, Tianjin, 300071 People’s Republic of China

**Keywords:** SnO_2_, Nanostructures, Anode, Li-ion battery, Na-ion battery

## Abstract

It is urgent to solve the problems of the dramatic volume expansion and pulverization of SnO_2_ anodes during cycling process in battery systems. To address this issue, we design a hybrid structure of N-doped carbon fibers@SnO_2_ nanoflowers (NC@SnO_2_) to overcome it in this work. The hybrid NC@SnO_2_ is synthesized through the hydrothermal growth of SnO_2_ nanoflowers on the surface of N-doped carbon fibers obtained by electrospinning. The NC is introduced not only to provide a support framework in guiding the growth of the SnO_2_ nanoflowers and prevent the flower-like structures from agglomeration, but also serve as a conductive network to accelerate electronic transmission along one-dimensional structure effectively. When the hybrid NC@SnO_2_ was served as anode, it exhibits a high discharge capacity of 750 mAh g^−1^ at 1 A g^−1^ after 100 cycles in Li-ion battery and 270 mAh g^−1^ at 100 mA g^−1^ for 100 cycles in Na-ion battery, respectively.

**Electronic supplementary material:**

The online version of this article (10.1007/s40820-017-0172-2) contains supplementary material, which is available to authorized users.

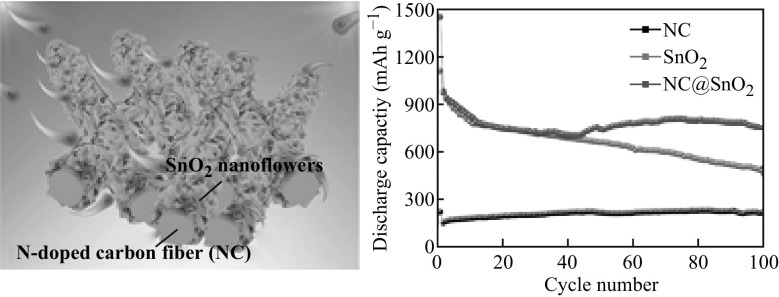

## Highlights

- A hybrid structure of SnO_2_ nanoflowers grown on N-doped carbon nanofibers (NC@SnO_2_) was successfully constructed.
- N-doped carbon nanofiber accelerates the migration of Li^+^/Na^+^ ions and guides the growth of the SnO_2_ nanoflowers.
- NC@SnO_2_ electrode reveals excellent energy storage performance for Li- and Na-ion batteries.

## Introduction

With severe resource constraints and global environmental problems, it is necessary to develop highly efficient energy storage systems to reduce the use of fossil fuels [1–5]. Nowadays, lithium- and sodium-ion batteries (LIBs and SIBs) have attracted widespread attention all over the world [6–8]. LIBs have been extensively applied in portable electronic equipment and electric vehicles (EVs) and intelligent power grids because of their outstanding characteristics of high energy density, no memory effect, and small self-discharge [9, 10]. Recently, owing to the lack of lithium resources and the similar chemical property of Na^+^ to Li^+^, SIBs have also received increasing attention [11, 12]. As one of the important parts for LIBs or SIBs, the high-performance electrode materials are urgently needed for next-generation battery systems.

As one of the typical transition-metal oxides (TMOs), tin dioxide (SnO_2_) is widely concerned to be promising electrode materials owing to its non-toxicity, low cost, high theoretical capacity, and outstanding electrochemical performance [13–15]. Nevertheless, it is similar to the shortcomings of other oxide materials during cycling processes that SnO_2_ endures the dramatic volume change. This would lead to the capacity decay and poor cycling performance [16–18]. To improve the electrochemical performance of SnO_2_, nanostructured SnO_2_ is employed to reduce the volume variation of SnO_2_ during the charge/discharge process [19–21]. However, it is easily agglomerated for nanostructured SnO_2_ to reduce the specific surface area of the active materials, leading to the attenuation of energy storage. To overcome this problem, a great deal of SnO_2_/carbon composites has been designed to maintain the structural stability of electrodes and improve the electrical conductivity of composites [22–24]. In addition, the N-doped carbon composite materials are considered to enhance the electrical conductivity and accelerate the reaction speed of the SnO_2_ composites, and increase defect sites for the efficient storage of lithium/sodium ions [25–27].

In this work, we synthesized a hybrid structure of N-doped carbon fibers@SnO_2_ nanoflowers (NC@SnO_2_) by electrospinning/hydrothermal methods. When they are used as an anode material in LIBs and SIBs, the as-prepared NC@SnO_2_ hybrid material displayed excellent electrochemical properties. The high discharge capacity reached 750 mAh g^−1^ at a current density of 1 A g^−1^ after 100 cycles in LIBs. Meanwhile, a reversible discharge capacity of 270 mAh g^−1^ was achieved at a current density of 100 mA g^−1^ after 100 cycles in SIBs.

## Experimental Section

### Synthesis of SnO_2_, N-doped Carbon, and NC@SnO_2_

All chemical reagents were purchased and used without further treatment. The synthesis of SnO_2_ nanoflowers was carried out according to the previous literature [28]. The N-doped carbon (NC) nanofibers were synthesized by electrospinning as follows: 0.6 g polyacrylonitrile (PAN, Sigma-Aldrich Co., Ltd. USA) was firstly added into 7 g *N*, *N*-dimethylformamide (DMF, Sinopharm Chemical Reagent Co., Ltd., China). Then, the above solution was poured into 10-mL plastic syringe and followed by electrospinning. The NC nanofibers were finally obtained via annealing the precursor at 600 °C in Ar atmosphere. To synthesize NC@SnO_2_, 4 mmol tin(II) chloride dihydrate (SnCl_2_·2H_2_O, Xilong Chemical Co., Ltd., China) and 8 mmol sodium citrate (Na_3_C_6_H_5_O_72_·H_2_O, Tianjin Hengxing Chemical Reagent Manufacturing Co., Ltd., China) were firstly dissolved into the mixed solvent of 15 mL ethanol and 15 mL water. After stirring for 30 min, 80 mg NC nanofibers were introduced into the above blend solution. Subsequently, the mixture solution was put into a Teflon-lined stainless steel autoclave at 180 °C for 12 h after continuous ultrasound for 30 min. The precursor samples were taken out the autoclave after the end of the reaction and ultrasonic cleaning with deionized water and ethanol. Finally, the NC@SnO_2_ samples were obtained with annealing at 500 °C for 3 h in Ar gas.

### Material Characterizations

The X-ray diffraction (XRD) of the samples was conducted with a Shimadzu XRD-6000 instrument, and the morphologies and structural features of the samples were characterized by scanning electron microscopy (SEM, Hitachi S4800) and transmission electron microscopy (TEM; JEOL 2010 with an accelerating voltage of 200 kV). The thermogravimetric analysis (TGA) of the powder sample was surveyed with a WCT-1D instrument (BOIF, China) in air atmosphere from 30 to 800 °C. Brunauer–Emmett–Teller (BET) of the sample was performed with the adsorption of N_2_ with a nova 2000 e volumetric adsorption analyzer (Kangta, USA), The element composition and chemical bonds of the sample were detected by X-ray photoelectron spectroscopy (XPS, Thermo Scientific Escalab 250Xi, USA). Raman spectra of the samples were conducted by utilizing micro-Raman spectrometer (LabRAM HR Evolution, HORIBA).

### Electrochemical Measurements

The working electrodes of LIBs and SIBs were fabricated by using 80 wt% of active materials (NC@SnO_2_, SnO_2_, and NC), 10 wt% of acetylene black, and 10 wt% of carboxymethylcellulose sodium (CMC). The mixture was uniformly distributed in the deionized water and ethanol and coated on the copper foil which dried at 60 °C in a vacuum drying oven for a day. CR2025-type coin half-batteries of as-prepared electrodes were assembled in the glove box with water and oxygen content of less than 0.5 ppm. The microporous polypropylene (Celgard 2400) and glass microfiber filter membranes (Whatman, Grade GF/A) were utilized as a separator of LIBs and SIBs, respectively. And corresponding metal plates were used as the counter electrodes of batteries. The electrolyte of LIBs was composed of 1.0 M of LiPF_6_ solution which mixed ethylene carbonate (EC) and dimethyl carbonate (DMC) with 1:1 in volume, and the electrolyte of SIBs was constituted by 1.0 M of NaClO_4_ solution which mixed EC with DMC (1:1 in volume), accompanied with 5% fluoroethylene carbonate (FEC) of additive agent. The electrochemical property and cyclic voltammetry measurement of LIBs and SIBs were performed with Neware Battery Testing System and CHI 660C Electrochemical Workstation, respectively.

## Results and Discussion

**Fig. 1:**
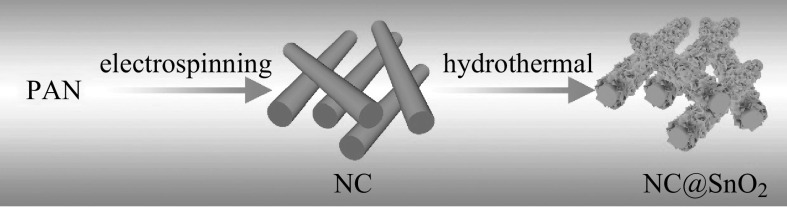
Schematic diagram for synthetic process of NC@SnO_2_. (Color figure online)

**Fig. 2:**
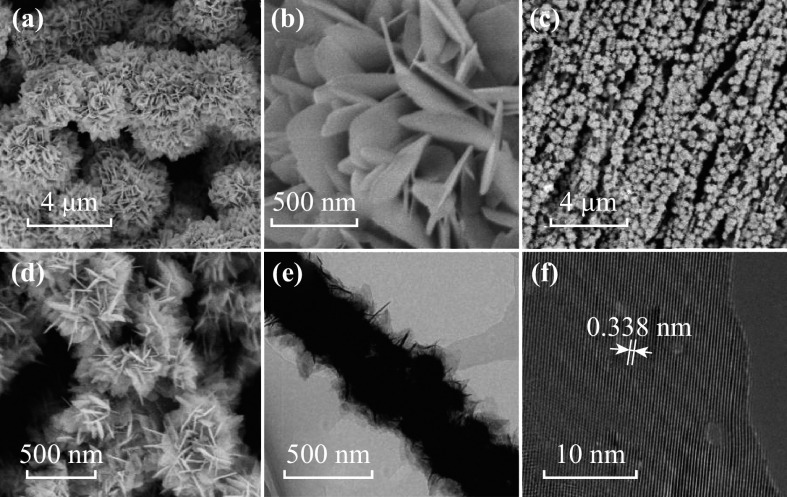
**a**, **b** SEM images of SnO_2_. **c**, **d** SEM images and **e**, **f**  TEM images of NC@SnO_2_

The synthetic process of NC@SnO_2_ is schematically shown in Fig. 1. Firstly, NC nanofibers were synthesized by the electrospinning method using PAN as the precursor, followed by heat treatment. Subsequently, SnO_2_ nanoflowers were grown on the NC nanofibers by hydrothermal method. The morphology and structures of the samples were characterized by SEM and TEM, as displayed in Fig. 2. Figure 2a, b displays that SnO_2_ sample is composed of agglomerated nanoflowers, which are assembled by nanosheets. When the NC nanofibers (Fig. S1) are introduced and used as the core for the hybrid structure, the SnO_2_ nanoflowers could grow on the surface of NC nanofibers, as shown in SEM images (Fig. 2c, d). Compared to SnO_2_ nanoflowers, the NC@SnO_2_ composite materials are formed by thinner nanosheets and better dispersed. In addition, the TEM image (Fig. 2e) of NC@SnO_2_ further indicates that one fiber is completely covered with the thin nanosheets. The high-resolution TEM image (Fig. 2f) indicates that the SnO_2_ nanosheet is well crystalline and has a lattice plane (110) with a crystal lattice distance of 0.338 nm.

**Fig. 3:**
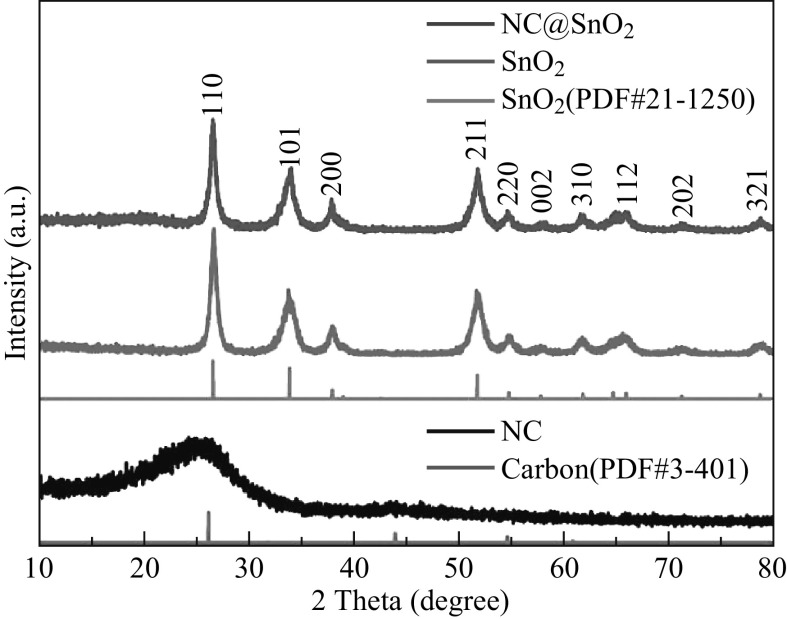
XRD patterns of NC@SnO_2_, SnO_2_, and NC. (Color figure online)

The crystal structures of NC@SnO_2_, SnO_2_, and NC materials were analyzed by XRD. From Fig. 3, one can observe that the diffraction peaks of NC@SnO_2_ and SnO_2_ are well consistent with the standard card (JCPDS card No. 21-1250), and the 2*θ* values of main peaks centered at 26.58, 33.88, 37.95, 51.75, and 54.76 are corresponded to the lattice planes of tetragonal SnO_2_ (110), (101), (200), (211), and (220), respectively. The diffraction peaks of NC are in accordance with the standard card (JCPDS card No. 3-401), and the 2*θ* values 26.60 and 54.79 are corresponded to the lattice planes of hexagonal carbon (006) and (0012), respectively. Nevertheless, the peak of carbon for the NC@SnO_2_ is not clearly observed. It is possible that the NC nanofibers were completely covered by the SnO_2_ nanoflowers, which make the carbon peaks disappear in NC@SnO_2_. The Raman spectrum of NC@SnO_2_ (Fig. S2) indicates that the two peaks at ~ 1350 and 1580 cm^−1^ are corresponded to the D peak of disorder carbon and the G peak of graphitic carbon. The relative intensity (*I*_D_/*I*_G_ ≈ 1.397 > 1) indicates that there exist mass defects caused by the N-doping in NC@SnO_2_ [29–31]. Additionally, the TGA curve of NC@SnO_2_ (Fig. S3) indicates that the lost weight of the sample appears in the range of 400–800 °C and the weight retention of SnO_2_ is confirmed to be about 67.81%.

**Fig. 4:**
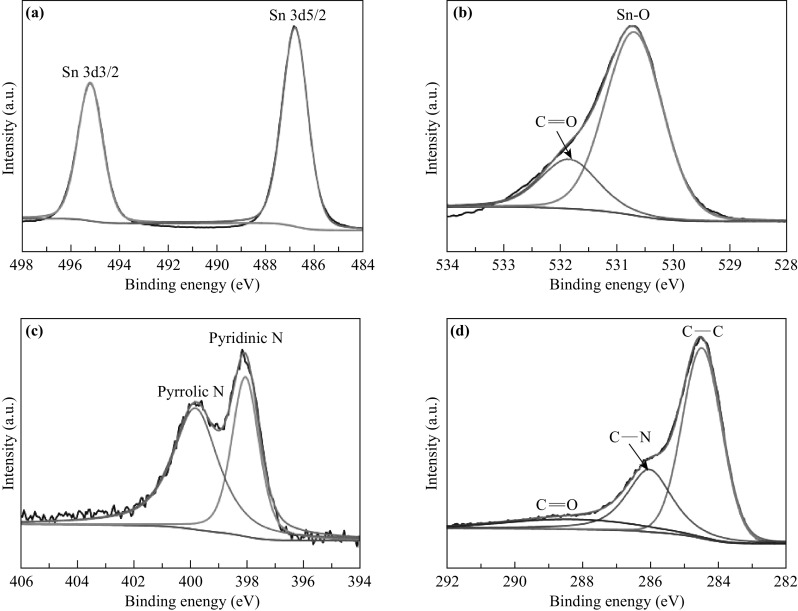
XPS spectra of the NC@SnO_2_: **a** Sn 3d, **b** O 1s, **c** N 1s, and **d** C 1s. (Color figure online)

The BET was utilized to confirm the surface information of the materials. Figure S4a, b shows the nitrogen adsorption–desorption isotherms of NC@SnO_2_ and SnO_2_ materials. The surface areas of NC@SnO_2_ and SnO_2_ are 45.59 and 37.01 cm^3^ g^−1^, respectively. Meanwhile, the pore-size distribution curves (Figs. S4c, d) indicate that the NC@SnO_2_ and SnO_2_ have the average pores of 3.74 and 2.56 nm, respectively. The larger specific surface area and pore size of NC@SnO_2_ are beneficial to the storage and transport of lithium/sodium ions. Moreover, the chemical component and surface electronic states of the NC@SnO_2_ material were further surveyed by XPS, and all peaks of these elements Sn, O, N, and C are observed as shown in Fig. S5 [32]. The high-resolution spectra of Sn 3d, O 1s, N 1s, and C 1s were recorded as shown in Fig. 4. The peaks of the Sn 1s (Fig. 4a) could be resolved into 486.78 and 495.12 eV, which are assigned to the binding energies of Sn 3d_5/2_ and Sn 3d_3/2_ of SnO_2_ [33]. The peaks of the O 1s (Fig. 4b) can be divided into two peaks of 530.61 and 531.36 eV and corresponded to Sn–O and C=O, respectively [34, 35]. The signal of N 1s could be obviously divided into two peaks of 398.00 and 399.76 eV (Fig. 4c), which well accorded with the binding energies of pyridinic N and pyrrolic N [36], it is verified the existence of nitrogen in NC@SnO_2_. Additionally, the peaks of the C 1s (Fig. 4d) could be resolved into three binding energies. The peak located at 285.89 eV corresponding to C–N bond can further confirm the presence of nitrogen in NC@SnO_2_ [37], and the other peaks of 284.42 and 288.54 eV are accorded with the binding energies of C–C and C=O, respectively [38].

**Fig. 5:**
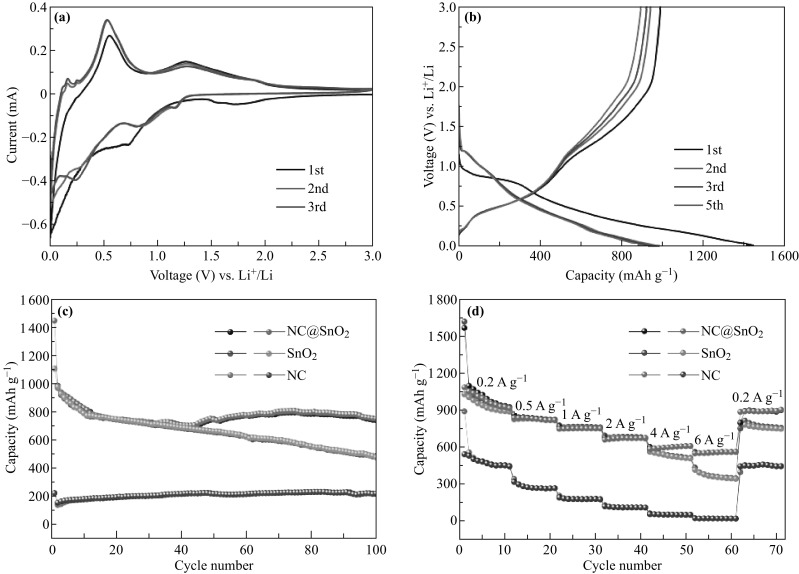
Electrochemical performance for LIBs: **a** Cyclic voltammetry curves of NC@SnO_2_ at 0.1 mV s^−1^. **b** Charge–discharge voltage profiles of NC@SnO_2_. **c** Cycling performances of NC@SnO_2_, SnO_2_, and NC at 1 A g^−1^. **d** Rate capabilities of NC@SnO_2_, SnO_2_, and NC. (Color figure online)

The NC@SnO_2_ was investigated as anode material for LIBs. SnO_2_ and NC nanofiber were also conducted for comparison. The CV curves of NC@SnO_2_ between 0.001 and 3.0 V vs Li^+^/Li at scan rate of 0.1 mV s^−1^ are shown in Fig. 5a, and it can be observed that there exists a subtle distinction in the first three cycles. The reduction peaks are found at the scope of 1.5–1.8 and 0.5–1.0 V at the first curve. They are attributed to the conversion process from SnO_2_ to Sn (Eq. 1) and the formation of SEI films, respectively [39, 40]. The peak below 0.5 V is associated with the lithiation (Li_x_Sn) of Sn (Eq. 2) [41]. In the following two CV curves, the reduction peaks at about 1.2, 0.8, and 0.3 V are associated with the reversible conversion reaction of SnO_2_ and alloy–dealloy reaction of Sn [42]. The oxidation peaks of about 0.8, 1.2 V and the small oxidation peak of 2.1 V are derived from the delithiation reaction of Li_x_Sn (Eq. 3) and reversible changing reaction of Sn between SnO and SnO_2_ (Eq. 4), respectively [42]. The Li^+^ de/intercalation of conversion process is described as follows,

1 $${\text{SnO}}_{2} + 4{\text{Li}}^{ + } + 4{\text{e}}^{ - } \to {\text{Sn}} + 2{\text{Li}}_{2} {\text{O,}}$$

2 $${\text{Sn}} + {\text{xLi}}^{ + } + {\text{xe}}^{ - } \to {\text{Li}}_{\text{x}} {\text{Sn }}\left( {0 \, < {\text{ x }} < 4.4} \right),$$

3 $${\text{Li}}_{\text{x}} {\text{Sn}} \to {\text{Sn}} + {\text{xLi}}^{ + } + {\text{xe}}^{ - } \left( {0 \, < {\text{ x }} < 4.4} \right),$$

4 $${\text{Sn}}/{\text{SnO}} + {\text{Li}}_{2} {\text{O}} \to {\text{SnO}}/{\text{SnO}}_{2} + 2{\text{Li}}^{ + } + 2{\text{e}}^{ - } .$$

The charge/discharge profiles of NC@SnO_2_ at the 1st, 2nd, 3rd, and 5th cycle were displayed at in Fig. 5b. The voltage platforms of charge–discharge can be observed to be consistent with the oxidation–reduction peaks of above CV curves. The initial discharge–charge capacities of NC@SnO_2_ are 1463.6 and 1009.8 mAh g^−1^, respectively. And the low initial coulombic efficiency of 67.0% may be associated with the formation of SEI film and the irreversible reactions of SnO_2_ material in the first cycle [32, 43]. The cycling performance of NC@SnO_2_, SnO_2_, and NC is shown in Fig. 5c. The discharge capacity of NC@SnO_2_ is about 750 mAh g^−1^ at 1 A g^−1^ after 100 cycles, while the discharge capacities of SnO_2_ and NC only remain 480 and 220 mAh g^−1^, respectively. In Fig. 5d, one can see that the average capacities of NC@SnO_2_ are about 1100, 850, 763, 684, 615, 568, and 905 mAh g^−1^ at different current densities of 0.2, 0.5, 1, 2, 4, 6, and 0.2 A g^−1^, respectively. However, the average capacities of SnO_2_ are only about 966, 842, 765, 685, 525, 370, and 770 mAh g^−1^ at 0.2, 0.5, 1, 2, 4, 6, and 0.2 A g^−1^, respectively. And the NC electrode exhibits the capacities less than 550 mAh g^−1^ at various current densities.

**Fig. 6:**
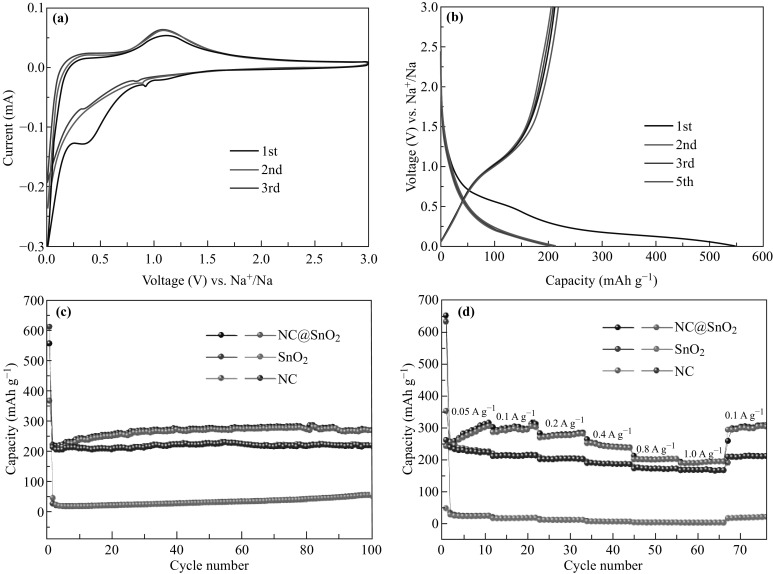
Electrochemical performance for SIBs: **a** Cyclic voltammetry curves of NC@SnO_2_ at 0.1 mV s^−1^. **b** Charge–discharge voltage profiles of NC@SnO_2_. **c** Cycling performances of NC@SnO_2_, SnO_2_, and NC at 100 mA g^−1^. **d** Rate capabilities of NC@SnO_2_, SnO_2_, and NC. (Color figure online)

The electrochemical property of NC@SnO_2_ was further investigated in SIBs. Figure 6a displays the CV curves of NC@SnO_2_ in the voltage range from 0.001 to 3.0 V vs Na^+^/Na at scan rate of 0.1 mV s^−1^. The obvious slope of 0.5–1.0 V is ascribed to the generation of the SEI film and the irreversible reactions between SnO_2_ with sodium ions to generate Na_x_Sn alloys in the first cycle [22]. In the initial three cycles, the two reduction peaks at about 1.0 and 0.3 V correspond to the insertion of sodium ions with the formation of Sn and Na_x_Sn, respectively. And the oxidation peak at 1.25 V corresponds to the de-intercalation of sodium ions [44, 45]. The reversible reaction of SnO_2_ with sodium ions to the production of Na_2_O and Na_x_Sn in the charge–discharge process can be represented as follows [44],

5 $${\text{SnO}}_{2} + 4{\text{Na}}^{ + } + 4{\text{e}}^{ - } \leftrightarrow {\text{Sn}} + 2{\text{Na}}_{2} {\text{O,}}$$

6 $${\text{Sn}} + {\text{xNa}}^{ + } + {\text{xe}}^{ - } \leftrightarrow {\text{Na}}_{\text{x}} {\text{Sn }}\left( {0 \, < {\text{ x }} < 4} \right).$$

Figure 6b displays the discharge/charge capacities of 555.7/212.5 mAh g^−1^ in the first charge/discharge cycle, respectively, with a coulombic efficiency of 38.2%. The low coulombic efficiency can be attributed to the formation of SEI film, and the irreversible reaction of SnO_2_ with sodium ion to form Na_x_Sn alloys in the first discharge process [46, 47]. In this work, the SnO_2_ and NC electrodes are used as a reference. In Fig. 6c, one can see that the discharge capacity of NC@SnO_2_ is about 270 mAh g^−1^, compared with 55 and 220 mAh g^−1^ of SnO_2_ and NC at 100 mA g^−1^ after 100 cycles. The rate performances for the three electrodes were also studied as shown in Fig. 6d. When the current densities were set at 0.05, 0.1, 0.2, 0.4, 0.8, 1, and 0.1 A g^−1^, the NC@SnO_2_ electrode exhibits the discharge capacities of about 295, 300, 280, 247, 202, 193, and 300 mAh g^−1^, respectively. These results are better than those of SnO_2_ and NC electrodes.

**Fig. 7:**
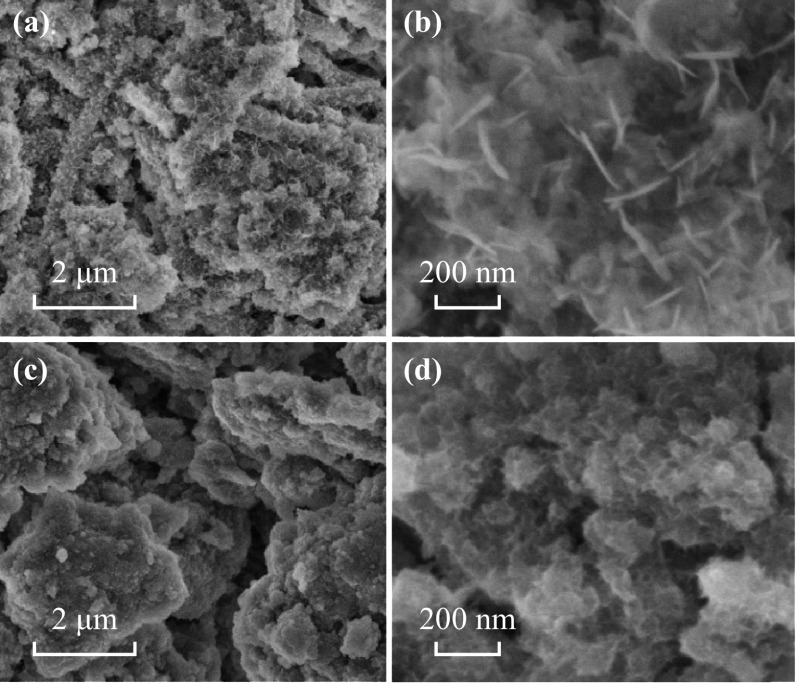
SEM images of the electrodes after cycles for the Li-ion batteries: **a**, **b** NC@SnO_2_ and **c**, **d** SnO_2_

To further demonstrate the structural stability of hybrid NC@SnO_2_, the SEM images of electrodes after about 75 cycles are given in Figs. 7 and S6. The SEM images of NC@SnO_2_ and SnO_2_ electrodes as anode for LIBs after cycling are shown in Fig. 7. Compared to the SEM images of the SnO_2_ electrodes (Fig. 7c, d), we can observe the obvious NC could be retained, and the SnO_2_ nanoflowers are not completely collapsed as shown in Fig. 7a, b. It demonstrates that the hybrid NC@SnO_2_ electrodes have the better cycle performance and rate capability than those of SnO_2_ electrodes in the LIBs due to the more stable structure of hybrid NC@SnO_2_ material. We also investigated the structural change of both NC@SnO_2_ and SnO_2_ electrodes for SIBs. As shown in the SEM images of NC@SnO_2_ electrode after cycling (Fig. S6a), the network structure of the NC could still be observed and no obvious reunion in comparison with the SnO_2_ electrode (Fig. S6c). However, SnO_2_ nanoflowers are completely collapsed in both NC@SnO_2_ and SnO_2_ electrodes, as shown in the high-magnification SEM images (Fig. S6b, d). This is because that the formation of Na–Sn alloy with enormous volume changes makes the pulverization of SnO_2_ material upon repetitive cycling [20]. These results indicated that NC nanofibers of the NC@SnO_2_ electrode can not only provide a conductive network, but also prevent the aggregation and pulverization of the SnO_2_ nanoflowers during the charge and discharge process.

## Conclusion

In summary, we have successfully prepared a hybrid structure of NC@SnO_2_ by electrospinning/hydrothermal methods. The NC nanofibers of the hybrid NC@SnO_2_ can prevent the agglomeration of SnO_2_ nanoflowers and effectively accelerate the transition of Li^+^/Na^+^ ion to promote the rate capability. Moreover, the structure can make more surface of the nanoflower exposed and buffer the volume expansion of SnO_2_ to enhance discharge capacity and cycling performance during cycling process. In addition, the hybrid NC@SnO_2_ could deliver a discharge capacity of 750 mAh g^−1^ after 100 cycles at 1 A g^−1^ for Li-ion battery and 270 mAh g^−1^ after 100 cycles at 100 mA g^−1^ for Na-ion battery.

## Electronic supplementary material

Below is the link to the electronic supplementary material.

Supplementary material 1 (PDF 680 kb)
