# Supplementary material for: Growth of SnO2 Nanoflowers on N-doped Carbon Nanofibers as Anode for Li- and Na-ion Batteries
Source: Nanomicro Lett. 2017 Dec 8;10(2):21. doi: 10.1007/s40820-017-0172-2 (PMC6199068; doi:10.1007/s40820-017-0172-2)
Supplement: Supplementary file 1 — Supplementary material 1 (PDF 680 kb) [file 40820_2017_172_MOESM1_ESM.pdf]

Supporting Information for

Growth of SnO_2 Nanoflowers on N-doped Carbon Nanofibers as Anode for Li- and Na-ion Batteries

Jiaojiao Liang[1, #], Chaochun Yuan[2, #], Huanhuan Li[2], Kai Fan[1], Zengxi Wei[1], Hanqi Sun[1], Jianmin Ma[1, 3, *]

[1]School of Physics and Electronics, Hunan University, Changsha 410082, People's Republic of China

[2]Automotive Engineering Research Institute, Jiangsu University, Zhenjiang 212013, People's Republic of China

[3]Key Laboratory of Advanced Energy Materials Chemistry (Ministry of Education), Nankai University, Tianjin 300071, People's Republic of China

[#]The two authors have equal contribution to this work.

*Corresponding author. E-mail: nanoelechem@hnu.edu.cn

Figures

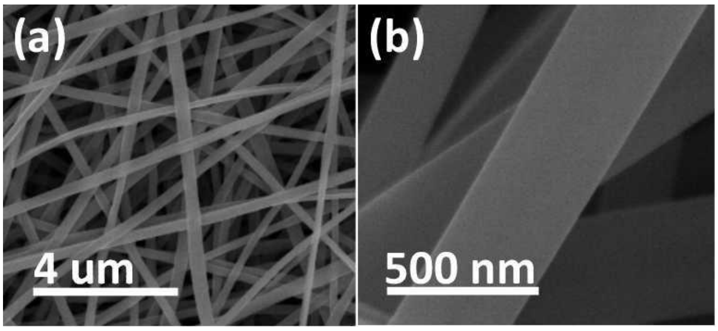

Fig. S1 a, b Low and high magnification SEM images of NC nanofibers

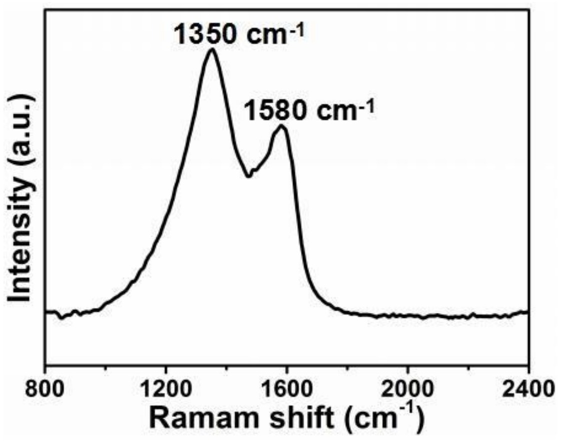

Fig. S2 Raman spectrum of NC@SnO_2

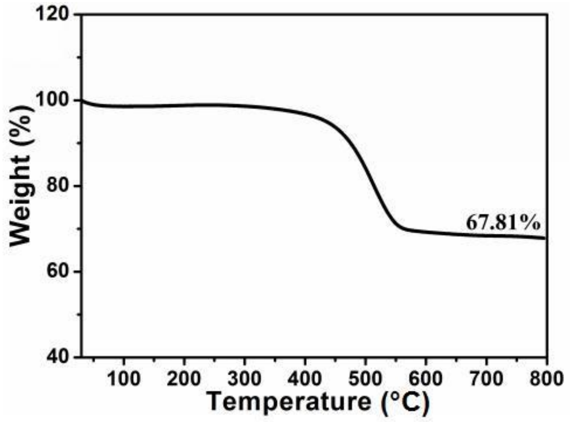

Fig. S3 TGA curve of NC@SnO_2

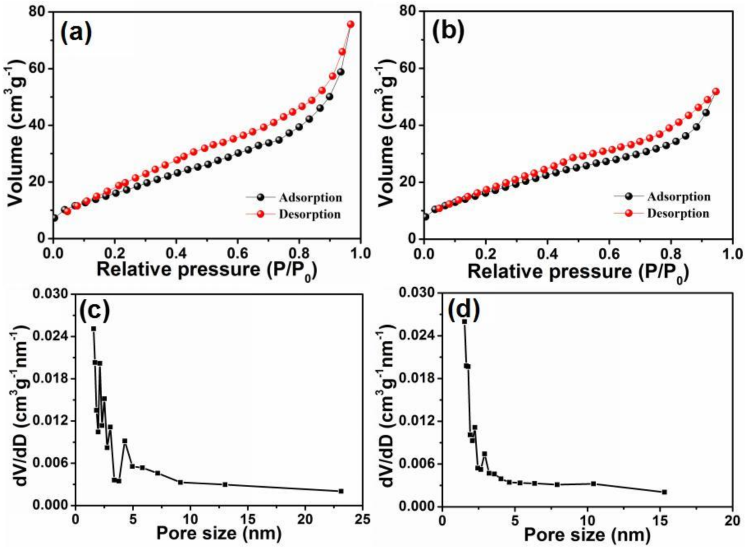

Fig. S4 a, b Nitrogen adsorption-desorption isotherms and **c, d** the pore-size distribution curves of NC@SnO_2 and SnO_2

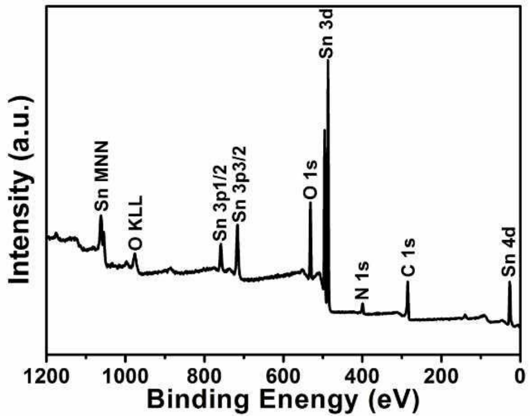

Fig.re S5 XPS spectrum of $NC@SnO_2$

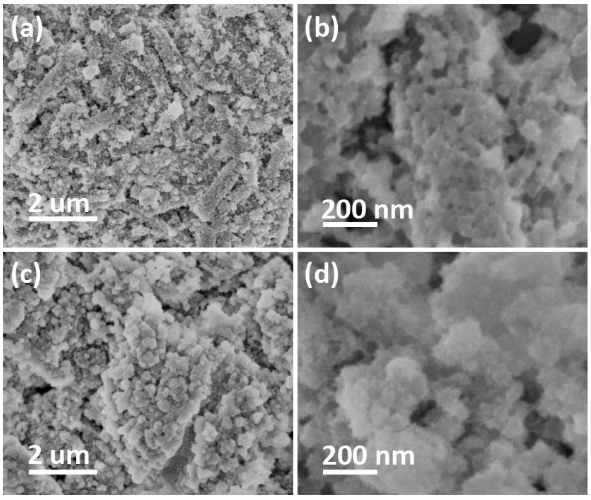

Fig. S6 SEM images of the electrodes after cycling for the NIBs: **a, b** $NC@SnO_2$; **c, d** SnO_2
